# Nonadherence to Self-Care Practices, Antihypertensive Medications, and Associated Factors among Hypertensive Patients in a Follow-up Clinic at Asella Referral and Teaching Hospital, Ethiopia: A Cross-Sectional Study

**DOI:** 10.1155/2021/7359318

**Published:** 2021-10-31

**Authors:** Addisu Dabi Wake, Techane Sisay Tuji, Addisu Tadesse Sime, Mekuria Tesfaye Mekonnin, Taju Mohamed Taji, Alfia Abdurahaman Hussein

**Affiliations:** ^1^Nursing Department, College of Health Sciences, Arsi University, Asella, Ethiopia; ^2^Sheno Health Center, Sheno, Oromia Regional State, Ethiopia; ^3^Burka Dhintu Woreda Health Office, Tayfe, Oromia Regional State, Ethiopia; ^4^Abomsa Hospital, Abomsa, Oromia Regional State, Ethiopia; ^5^Shashamane General Hospital, Shashamane, Oromia Regional State, Ethiopia

## Abstract

**Background:**

Hypertension is one of the most common noncommunicable diseases affecting several individuals globally. However, the level of nonadherence to self-care practices, antihypertensive medications, and associated factors among hypertensive patients in a follow-up clinic at Asella Referral and Teaching Hospital is unknown.

**Objective:**

To assess the level of nonadherence to self-care practices, antihypertensive medications, and associated factors among hypertensive patients in a follow-up clinic at Asella Referral and Teaching Hospital, Arsi Zone, Oromia Regional State, Ethiopia, in 2020.

**Methods:**

An institution-based cross-sectional survey was conducted on 115 hypertensive patients who visited the follow-up clinic at Asella Referral and Teaching Hospital from December 24, 2020, to January 15, 2021. Data were entered into EpiData version 4.2.0.0 and exported to SPSS version 21.0 for statistical analysis. Binary and multivariable logistic regression analysis was used to assess the presence of statistical association between dependent and independent variables.

**Results:**

A total of 115 hypertensive patients were enrolled into the study, giving a response rate of 98.29%. The mean age of the study participants was 55.17 years (SD = 17.986). More than half of them (59 (51.3%)) were females. More than half of them (58 (50.4%)) were married. Nearly two-thirds of them (79 (68.7%)) had formal education. The level of nonadherence to self-care practices was 67.0% (*n* = 77, 95% CI: 60.0, 75.7). Meanwhile, the patient's level of nonadherence to antihypertensive medications was 16.5% (*n* = 19, 95% CI: 10.4, 24.3). The multivariable logistic regression analysis showed that age >45 years (AOR = 2.89, 95% CI: 1.16, 7.18), having no formal education (AOR = 1.67, 95% CI: 1.32, 3.74), and having ≤5 years' duration since diagnoses of hypertension (AOR = 1.56, 95% CI: 1.07, 3.25) were factors significantly associated with nonadherence to self-care practices. Being male (AOR = 2.09, 95% CI: 1.93, 9.59), being married (AOR = 4.22, 95% CI: 1.29, 13.76), and having an average monthly income of ≤2500 ETB (AOR = 1.58, 95% CI: 1.09, 7.08) were factors significantly associated with nonadherence to medications.

**Conclusion:**

In the present study, the level of both nonadherence to self-care practices and antihypertensive medications was relatively high. There is a need to initiate programs that could create awareness about adherence to self-care practices and antihypertensive medications among hypertensive patients to improve their level of adherence.

## 1. Background

Hypertension is a higher systemic blood pressure (BP) that leads to an obvious upsurge of cardiovascular risk [1]. Resistant hypertension is linked to an augmented risk of cardiovascular disease (CVD) [2]. The relationship between BP and the risk of CVD is not depending on other risk factors [3]. CVDs were the main cause of morbidity and mortality in Ethiopia [4]. Hypertension is a worldwide community health problem [5]. It is a community health problem and is not well controlled [6]. It is upsetting approximately 1 billion individuals worldwide [7]. It remains as a giant community health and economic burden worldwide [8]. It is one of the world's most fatal diseases [9].

The magnitude of hypertension is growing in Africa. However, people were not aware of their situation [10]. In Africa, there were poor awareness, treatment, and control of hypertension [11]. Hypertension is a substantial public health test globally and this condition should acquire high priority [12]. Supportive efforts are instantaneously compulsory to fight the developing hypertension burden [13]. BP dropping markedly declines vascular risk and comorbidities [14].

The probable causes of uncontrolled BP were poor medication adherence, lack of information about hypertension, and side effects [15]. A steady medication adherence is a crucial factor to sustained BP control. Healthcare providers require understanding the main clues related to suboptimal adherence or nonadherence that can lead to poor outcomes [16]. In turn, nonadherence to antihypertensive medications was related to an augmented risk of stroke [17]. Low adherence to antihypertensive medications remains a population health burden. Knowing the barriers and determinants, factors of adherence to antihypertensive medication may benefit and recognize interventions to increase adherence [18].

The strategies to decrease BP in the high-risk population should be a national priority [2]. Healthy lifestyle recommendations play a significant role in controlling BP [19]. This is evidenced by adherence to a healthy lifestyle which is associated with a lower risk of hypertension [20]. When used effectively, lifestyle modifications have a significant role in the management of hypertension [21]. A long-term adherence to antihypertensive medication has verified the importance [22]. Furthermore, compliance with antihypertensive medications was found to be improved after providing education about hypertension and its related complications [23]. Counseling hypertensive individuals towards adherence to medication and the public with suitable information about hypertension management would improve the patient's level of adherence [24]. In addition to this, to decrease hypertension related morbidity and mortality, identifying individuals at risk of nonadherence to treatment and poor blood pressure control can lead to targeted interventions [25].

As explained overhead, the burden of hypertension is increasing worldwide. Gaining information concerning the level of nonadherence to self-care practices, medications, and associated factors will be used as integrated management in managing and controlling hypertension. Since the present study has determined the level of nonadherence to self-care practices, medications, and associated factors among hypertensive patients, the result would aware the hospitals, healthcare professionals, government stakeholders, and researchers.

## 2. Objectives

### 2.1. General Objective

The general objective is to assess the level of nonadherence to self-care practices, antihypertensive medications, and associated factors among hypertensive patients in a follow-up clinic at Asella Referral and Teaching Hospital, Arsi Zone, Oromia Regional State, Ethiopia, in 2020.

### 2.2. Specific Objectives

- To determine the level of nonadherence to self-care practices among hypertensive patients in follow-up clinics at Asella Referral and Teaching Hospital, Arsi Zone, Oromia Regional State, Ethiopia, in 2020
- To determine the level of nonadherence to antihypertensive medications among hypertensive patients in follow-up clinics at Asella Referral and Teaching Hospital, Arsi Zone, Oromia Regional State, Ethiopia, in 2020
- To identify factors associated with nonadherence to self-care practices among hypertensive patients in follow-up clinics at Asella Referral and Teaching Hospital, Arsi Zone, Oromia Regional State, Ethiopia, in 2020
- To identify factors associated with nonadherence to antihypertensive medications among hypertensive patients in follow-up clinics at Asella Referral and Teaching Hospital, Arsi Zone, Oromia Regional State, Ethiopia, in 2020

## 3. Methods

### 3.1. Study Area and Period

The study was conducted in Asella Referral and Teaching Hospital from December 24, 2020, to January 15, 2021. Asella Referral and Teaching Hospital is located in Asella town which is located about 175 KMs southeast from Addis Ababa, the capital city of Ethiopia [26].

### 3.2. Study Design

An institution-based cross-sectional survey was conducted at Asella Referral and Teaching Hospital.

### 3.3. Source Population

The source population was all hypertensive patients who visited the follow-up clinic at Asella Referral and Teaching Hospital.

### 3.4. Study Population

The study population was all hypertensive patients who visited the follow-up clinic at Asella Referral and Teaching Hospital and fulfilled the inclusion criteria.

### 3.5. Eligibility Criteria

#### 3.5.1. Inclusion Criteria

Inclusion criteria included all hypertensive patients aged ≥18 years.

#### 3.5.2. Exclusion Criteria

Hypertensive patients who were severely ill and physically unable to be interviewed at the time of data collection were excluded.

### 3.6. Sample Size Determination

Survey was conducted among hypertensive patients who visited the follow-up clinic at Asella Referral and Teaching Hospital. Since the survey was undertaken, all hypertensive patients attending the follow-up clinic at Asella Referral and Teaching Hospital were included in the study. The final sample size was 115 (*n* = 115).

### 3.7. Sampling Technique and Procedures

During the survey, the hypertensive patient was checked using the medical registration card. Afterwards, all hypertensive patients present on the day of the survey and who were willing to participate in the survey were included in the study.

### 3.8. Study Variables

#### 3.8.1. Dependent Variable

- Nonadherence to self-care practices
- Nonadherence to antihypertensive medications

#### 3.8.2. Independent Variables

Sociodemographic variables are gender, age, religion, ethnicity, marital status, educational level, occupation, and average monthly income. Health-related factors include comorbidity and duration since diagnosed hypertension.

### 3.9. Operational Definitions

Adherence to self-care practices is when the patient scored the mean or above mean score [27]. Nonadherence to self-care practices is when the patient scored below the mean score [27].

### 3.10. Data Collection Instrument

The questionnaire was adapted and developed from relevant literatures with modification fit to the local context [27–32]. During this, various experts were involved in it. The questionnaire was prepared in English and translated to Afan Oromo and finally translated back to English to maintain consistency.

### 3.11. Data Collection Procedures

A semistructured interviewer-administered questionnaire and patients' medical records review was used to collect data. Data were collected by four nurses with Bachelor of Science degree, and the study was supervised by one nurse with Master of Science degree.

### 3.12. Data Quality Control

The questionnaire was pretested on 5% of the sample size. Data collectors were trained for one day on the data collection instrument and data collection procedure. The reliability of the questionnaire was checked by reliability analysis and Cronbach's alpha value was 0.79 which suggested a reliable tool. During the data collection period, a close supervision was done by the supervisor.

### 3.13. Data Processing and Analysis

Data was checked, coded, and entered into EpiData version 4.2.0.0, and then it was exported to Statistical Package for the Social Sciences (SPSS) version 21.0 (IBM Corporation, North Castle Drive, Armonk, NY, USA) for statistical analysis. The outcome variable was dichotomized and coded as 0 and 1 representing adherence and nonadherence, respectively. Texts, tables, and figures were used to summarize the descriptive statistics.

Both bivariable and multivariable logistic regression analyses were carried out to find the variables associated with the dependent variables. Both the crude odds ratios (COR) and adjusted odds ratio (AOR) with the corresponding 95% confidence were determined to show the strength of the association. Finally, variables with *P* value <0.05 in the multivariable logistic regression were considered as statistically significant.

## 4. Results

### 4.1. Sociodemographic Characteristics of Study Participants

In the present study, a total of 115 hypertensive patients were enrolled into the study, giving a response rate of 98.29%. The mean age of the patients was 55.17 years (SD = 17.986), while the majority of the patients (72 (62.6%)) were aged >45 years. More than half of the patients (59 (51.3%)) were females. Regarding religion, 58 patients (50.4%) were Muslims. The majority of patients (80 (69.6%)) were Oromo by ethnic background. More than half of the patients (58 (50.4%)) were married. Nearly two-thirds of the patients (79 (68.7%)) had formal education (Table 1).

### 4.2. Health-Related Factors

Among the study participants, 75 (65.2%) were ≤5 years since diagnosis with hypertension. 52 (45.2%) of them have been on hypertensive treatment for four or more years. Regarding the number of types of medications, 108 (93.9%) of them took less than or equal to the two types of medications. Furthermore, 23 (20.0%) of them reported that they had comorbidities, such as diabetics (43.5%), stroke (30.5%), and chronic kidney disease (13.0%) (Table 2).

### 4.3. Adherence to the Components of Self-Care Practices

#### 4.3.1. Adherence to Dietary Modifications

Among the study participants, 50 (43.5%) of them reported that they had rarely included fruits, vegetables, grains, and beans in their diet after they were diagnosed with hypertension. Moreover, nearly more than half of them (58 (50.4%)) reported that they had rarely consumed foods that contain high saturated fat such as cheese, coconut oil, cotton seed oil, and mutton fat since being diagnosed. Furthermore, 66 (57.4%) of them never consumed spicy foods since they were diagnosed. 64 (55.7%) of them never consumed salt in their foods. 80 (69.6%) of them never read nutritional facts on food labels to compare the amount of sodium in the products (Table 3).

#### 4.3.2. Adherence to Exercise

The majority of the participants (98 (85.2%)) answered that they performed physical exercise. Regarding the frequency of performing physical exercise, 47 (48.0%) of them performed physical exercise more than three times per week. Walking (69.4%), jogging (25.5%), and cycling (5.1%) were the major types of exercise performed by these hypertensive patients. Regarding the duration of performing physical exercise, 52 (53.1%) of them reported less than 30 minutes (Table 4).

#### 4.3.3. Adherence to Cessation of Smoking

The majority of hypertensive patients (96 (83.5%)) never used tobacco. Among those cigarette smokers, 8 (42.1%) of them were still smokers and 6 (75%) of them tried to quit smoking (Table 5).

#### 4.3.4. Adherence to Moderation of Alcohol Consumption

The majority of hypertensive patients (98 (85.2%)) never consumed alcohol even on occasion. Most of them (97 (84.3%)) reported that they were advised to cut down drinking alcohol by relatives/friends/doctors/health workers (Table 6).

### 4.4. Level of Nonadherence to Self-Care Practices

**Figure 1:**
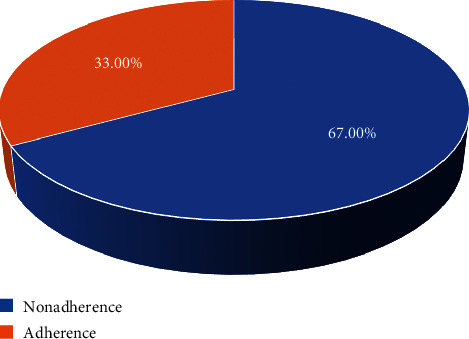
The level of nonadherence to self-care practices among hypertensive patients in a follow-up clinic at Asella Referral and Teaching Hospital, Asella Town, Arsi Zone, Oromia Regional State, Ethiopia, 2020 (*n* = 115).

The level of nonadherence to self-care practices was 67.0% (*n* = 77, 95% CI: 60.0, 75.7) (Figure 1).

### 4.5. Level of Nonadherence to Antihypertensive Medications

**Figure 2:**
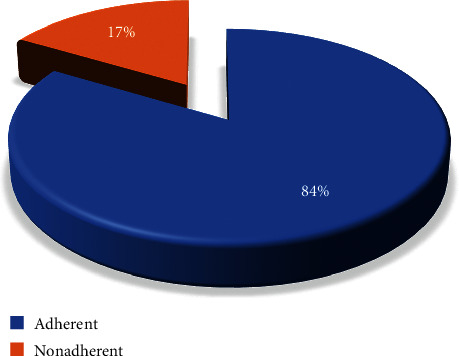
The level of nonadherence to antihypertensive medications among hypertensive patients in a follow-up clinic at Asella Referral and Teaching Hospital, Asella Town, Arsi Zone, Oromia Regional State, Ethiopia, 2020 (*n* = 115).

The patients' level of nonadherence to antihypertensive medications was 16.5% (*n* = 19, 95% CI: 10.4, 24.3) (Figure 2).

### 4.6. Factors Associated with Nonadherence to Self-Care Practices

To identify independent factors associated with nonadherence to self-care practices, gender, age, marital status, educational level, occupation, average monthly income, duration since diagnosis, and comorbidities were entered into both binary and multivariable logistic regression analyses. However, only age, educational level, and duration since diagnosis were factors significantly associated with nonadherence to self-care practices.

The odds ratio of having nonadherence to self-care practices among hypertensive patients who were aged >45 years was 2.89 times (AOR = 2.89, 95% CI: 1.16, 7.18) higher than hypertensive patients who were aged ≤45 years. Likewise, the likelihood of having nonadherence to self-care practices among hypertensive patients who had no formal education was 1.67 times (AOR = 1.67, 95% CI: 1.32, 3.74) more when compared with those who had a formal education. Those hypertensive patients who had ≤5 years' duration since diagnosis of hypertension were 1.56 times (AOR = 1.56, 95% CI: 1.07, 3.25) more likely to have nonadherence to self-care practices when compared to hypertensive patients who had >5 years' duration since diagnosis of hypertension (Table 7).

### 4.7. Factors Associated with Nonadherence to Antihypertensive Medications

Likewise, to identify independent factors associated with nonadherence to medications, gender, age, marital status, educational level, occupation, average monthly income, duration since diagnosis, and comorbidities were entered into both bivariable and multivariable logistic regression analyses. However, only gender, marital status, and average monthly income were factors significantly associated with nonadherence to antihypertensive medications.

The odds ratio of having nonadherence to antihypertensive medications among hypertensive patients who were male was 2.09 times (AOR = 2.09, 95% CI: 1.93, 9.59) higher than hypertensive patients who were female. Those hypertensive patients who were married were 4.22 times (AOR = 4.22, 95% CI: 1.29, 13.76) more likely to have nonadherence to medications when compared to hypertensive patients who were unmarried. Furthermore, the likelihood of having nonadherence to medications among hypertensive patients who had an average monthly income of ≤2500 ETB was 1.58 times (AOR = 1.58, 95% CI: 1.09, 7.08) more when compared with those who had a >2500 ETB average monthly income (Table 8).

## 5. Discussion

Ensuring hypertensive patients' adherence to medications and self-care practices to manage and prevent complications of hypertension remains a major challenge to public health globally. Poor adherence to treatment is a reason for uncontrolled hypertension, serious complications, and wastage of healthcare resources. The present study determined the level of nonadherence to self-care practices, antihypertensive medications, and associated factors among hypertensive patients in the follow-up clinic at Asella Referral and Teaching Hospital, Ethiopia.

### 5.1. Nonadherence to Self-Care Practices and Associated Factors

The patients' level of nonadherence to self-care practices was 67.0% (*n* = 77, 95% CI: 60.0, 75.7). The present study's finding was lower when compared with a study done in Addis Ababa, Ethiopia (77%) [27]. The variation might be due to the duration since the study was conducted, while the study of Addis Ababa was done in 2016. Within this long duration, there could be a lot of information dissemination through different sources such as mass media and newspapers. The present study's finding was also lower when compared with a study done in Dessie Referral Hospital, Ethiopia (76.4%) [33]. The possible justification could be the differences in sociodemographic characteristics of the study participants. However, the present study's finding was higher when compared with a study conducted in Harari Region, Ethiopia (37.9%) [34]. This might be due to the differences in sociodemographic characteristics of the study participants.

The odds ratio of having nonadherence to self-care practices among hypertensive patients who were aged >45 years was 2.89 times (AOR = 2.89, 95% CI: 1.16, 7.18) higher than hypertensive patients who were aged ≤45 years. The likelihood of having a nonadherence to self-care practices among hypertensive patients who had no formal education was 1.67 times (AOR = 1.67, 95% CI: 1.32, 3.74) more when compared with those who had a formal education. In fact, education has the power to create awareness for patients concerning disease seriousness. Those hypertensive patients who had ≤5 years' duration since diagnosis of hypertension were 1.56 times (AOR = 1.56, 95% CI: 1.07, 3.25) more likely to have nonadherence to self-care practices when compared to hypertensive patients who had >5 years' duration since diagnosis of hypertension.

### 5.2. Nonadherence to Antihypertensive Medication and Associated Factors

The patients' level of nonadherence to antihypertensive medications was 16.5% (*n* = 19, 95% CI: 10.4, 24.3). The present study's finding was also lower when compared with a study done in Nedjo, Ethiopia (68.6%) [30]. This might be the length since the study was conducted, which was from March 15 to May 5, 2015, for the study done in Nedjo. In fact, through this duration of time, there could be a change in the treatment protocol and there might be involvement of the mass media on the awareness and creation of the disease and pharmacologic treatment.

The present study's finding was lower when compared with a study done in Buea, Cameroon (67.7%) [28]. This might be due to the differences in the sociodemographic characteristics of the study participants. Besides, there are differences in the study setting, while the study done in Buea, Cameroon, was a community-based study. The present study's finding was also lower when compared with a multicenter study done in Ghana and Nigeria (66.7%) [31]. This might be due to the length of duration since the study was done, which was from April to September 2013 for the study done in Ghana and Nigeria. Besides, the differences in the sociodemographic characteristics of the study participants could play a significant role in this variation.

The present study's finding was also lower when compared with a study done in Kinshasa, Democratic Republic of Congo (54.2%) [35]. This might be due to the differences in sociodemographic characteristics of the study participants. The present study's finding was also lower when compared with a study done in Nigeria (45.8%) [36]. The present study's finding was also lower when compared with a study done in Brazil (71%) [29]. This might be due to the length of duration since the study was done, where the study of Brazil was done from November 2012 to April 2013. The present study's finding was consistent when compared with a study done in Korea (13.2%) [32].

The odds ratio of having nonadherence to medications among hypertensive patients who were male was 2.09 times (AOR = 2.09, 95% CI: 1.93, 9.59) higher than hypertensive patients who were female. Those hypertensive patients who were married were 4.22 times (AOR = 4.22, 95% CI: 1.29, 13.76) were more likely to have nonadherence to medications when compared to hypertensive patients who were unmarried. The likelihood of having nonadherence to medications among hypertensive patients who had an average monthly income of ≤2500 ETB was 1.58 times (AOR = 1.58, 95% CI: 1.09, 7.08) more when compared with those who had a >2500 ETB average monthly income. The present study's finding was supported by the study done in Nedjo, Ethiopia [30]. This might be due to the scarcity of money which could affect the expense of transport and accessibility to sources of information such as mass media and newspapers. Meanwhile, in turn, these could influence the level of adherence to antihypertensive medications.

## 6. Conclusion

In the present study, the hypertensive patients' level of nonadherence to self-care practices was 67.0%. Meanwhile, the patients' level of nonadherence to antihypertensive medications was 16.5%. The multivariable logistic regression analysis showed that age, educational level, and duration since diagnosis were factors significantly associated with nonadherence to self-care practices, whereas gender, marital status, and average monthly income were factors significantly associated with nonadherence to medications.

Moreover, the present study offers significant information to motivate the public for their health concerns. Ensuring hypertensive patients' adherence to self-care practices and antihypertensive medications to manage and prevent complications of hypertension is the foundation method. This is because nonadherence to self-care practices and medications is a principal reason for uncontrolled hypertension, serious complications, and wastage of healthcare resources. Thus, the findings of the present study also give a basis to support healthcare providers to consider hypertension management and encourage them to focus on it and design methods to address this problem. Finally, we suggest that there is a need to initiate programs that could create awareness about adherence to self-care practices and antihypertensive medications among hypertensive patients to improve their level of adherence.

### 6.1. Limitations of the Study

The present study had some limitations. The first limitation was the study design we have used, which was a cross-sectional survey. Another is there was a scarcity of the studies undertaken, which affects the discussion of various variables of the present study. Hopefully, the present study could minimize such challenges being baseline for the researchers who wish to discuss this topic in the future.

## Figures and Tables

**Table 1:** Sociodemographic characteristics of hypertensive patients in a follow-up clinic of Asella Referral and Teaching Hospital, Asella Town, Arsi Zone, Oromia Regional State, Ethiopia, 2020 (*n* = 115).

Variables	Category	Frequency	Percent
Gender	Male	56	48.7
	Female	59	51.3

Age	≤45 years	43	37.4
	>45 years	72	62.6

Religion	Orthodox	41	35.7
	Muslim	58	50.4
	Protestant	15	13.0
	Catholic	1	0.9

Ethnicity	Oromo	80	69.6
	Amhara	25	21.7
	Gurage	6	5.2
	Others	4	3.5

Marital status	Married	58	50.4
	Unmarried	57	49.6

Educational level	No formal education	36	31.3
	Formal education	79	68.7

Occupation	Employed	60	52.2
	Unemployed	55	47.8

Average monthly income	≤2500 ETB	73	63.5
	>2500 ETB	42	36.5

**Table 2:** Health related factors among hypertensive patients in a follow-up clinic of Asella Referral and Teaching Hospital, Asella Town, Arsi Zone, Oromia Regional State, Ethiopia, 2020 (*n* = 115).

Variables	Category	Frequency	Percent
Duration since diagnosis	≤5 years	75	65.2
	>5 years	40	34.8

Duration of antihypertensive treatment	Less than two years	16	13.9
	Two to four years	47	40.9
	Four or more years	52	45.2

Number of types of medications	≤Two	108	93.9
	>Two	7	6.1

Presence of comorbidities	Yes	23	20.0
	No	92	80.0

Diagnosed comorbidities (*n* = 23)	Diabetes mellitus	10	43.5
	Chronic kidney disease	3	13.0
	Stroke	5	21.7
	Chronic heart diseases	2	8.7

**Table 3:** Adherence to dietary modifications among hypertensive patients in a follow-up clinic of Asella Referral and Teaching Hospital, Asella Town, Arsi Zone, Oromia Regional State, Ethiopia, 2020 (*n* = 115).

Variables	Responses			
	Never	Rarely	Usually	Always
Included fruit, vegetables, grains, and beans in their diet after being diagnosed with hypertension	37 (32.2%)	50 (43.5%)	22 (19.1%)	6 (5.2%)
Consumed foods that contain high saturated fat	49 (42.6%)	58 (50.4%)	6 (5.2%)	2 (1.7%)
Consumed spicy foods since being diagnosed	66 (57.4%)	42 (36.5%)	3 (2.6%)	4 (3.5%)
Consumed salt in their food	64 (55.7%)	29 (25.2%)	12 (10.4%)	10 (8.7%)
Read nutritional facts on food labels to compare amount of sodium in products	80 (69.6%)	26 (22.6%)	4 (3.5%)	5 (4.3%)

**Table 4:** Adherence to exercise among hypertensive patients in a follow-up clinic of Asella Referral and Teaching Hospital, Asella Town, Arsi Zone, Oromia Regional State, Ethiopia, 2020 (*n* = 115).

Variables	Category	Frequency	Percent
Perform physical exercise (*N* = 115)	Yes	98	85.2
	No	17	14.8

How often do you exercise? (*N* = 98)	<Three times per week	30	30.6
	Three times per week	21	21.4
	>Three times per week	47	48.0

Types of exercise performed (*N* = 98)	Walking	68	69.4
	Jogging	25	25.5
	Cycling	5	5.1

Duration of exercise per session	<30 minutes	52	53.1
	>30 minutes	46	46.9

**Table 5:** Adherence to cessation of smoking among hypertensive patients in a follow-up clinic of Asella Referral and Teaching Hospital, Asella Town, Arsi Zone, Oromia Regional State, Ethiopia, 2020 (*n* = 115).

Variables	Category	Frequency	Percent
Ever used tobacco (*N* = 115)	Yes	19	16.5
	No	96	83.5

Still smoking cigarettes (*N* = 19)	Yes	8	42.1
	No	11	57.9

Ever tried to quit smoking (*N* = 8)	Yes	6	75.0
	No	2	25.0

**Table 6:** Adherence to moderation of alcohol consumption among hypertensive patients in a follow-up clinic of Asella Referral and Teaching Hospital, Asella Town, Arsi Zone, Oromia Regional State, Ethiopia, 2020 (*n* = 115).

Variables	Never	<1 month	Monthly	Weekly	Daily
How often do you have 8 drinks (men)/6 drinks (women) or more on occasion?	98 (85.2%)	8 (7.0%)	4 (3.5%)	4 (3.5%)	1 (0.9%)
How often in the last year have you not been able to remember what happened when drinking the night before?	112 (97.4%)	2 (1.7%)	0 (0)	1 (0.9%)	0 (0)
Has a relative/friend/doctor/health worker been concerned about your drinking or advised you to cut down on your drinking?	Yes		97 (84.3%)		
	No		18 (15.7%)		

**Table 7:** Bivariable and multivariable analysis of factors associated with nonadherence to self-care practices among hypertensive patients in a follow-up clinic at Asella Referral and Teaching Hospital, Asella Town, Arsi Zone, Oromia Regional State, Ethiopia, 2020.

Variables	Categories	Self-care practices		COR (95% CI)	AOR (95% CI)	*P* value
		Adherence	Nonadherence			
Gender	Male	20 (35.7%)	36(64.3%)	1	1	
	Female	18 (30.5%)	41(69.5%)	1.27(0.58, 2.76)	1.36(0.56, 3.20)	0.487

Age in years	≤45	21 (48.8%)	22(51.2%)	1	1	
	>45	17 (23.6%)	55 (76.4%)	3.09 (1.38, 6.93)	**2.89 (1.16, 7.18)**	**0.023**

Marital status	Unmarried	16 (28.1%)	41 (71.9%)	1.57 (0.72, 3.43)	1.76 (0.76, 4.11)	0.189
	Married	22 (37.9%)	36 (62.1%)	1	1	

Educational status	No formal education	9 (25%)	27 (75%)	1.74 (0.72, 4.20)	**1.67 (1.32, 3.74)**	**0.034**
	Formal education	29 (36.7%)	50 (63.3%)	1	1	

Occupation	Unemployed	20 (36.4%)	35 (63.6%)	0.75 (0.34, 1.63)	0.62 (0.22, 1.71)	0.352
	Employed	18 (30%)	42 (70%)	1	1	

Average monthly income	≤2500 ETB	22 (30.1%)	51 (69.9%)	1.43 (0.64, 3.17)	1.30 (0.49, 3.43)	0.591
	>2500 ETB	16 (38.1%)	26 (61.9%)	1	1	

Duration since diagnoses	≤5 years	23 (30.7%)	52 (69.3%)	1.36 (0.61, 3.04)	**1.56 (1.07, 3.25)**	**0.042**
	>5 years	15 (37.5%)	25 (62.5%)	1	1	

Presence of comorbidities	Yes	9 (39.1%)	14 (60.9%)	1	1	
	No	29 (31.5%)	63 (68.5%)	1.40 (0.54, 3.59)	1.61 (0.58, 4.49)	0.362

**Table 8:** Bivariable and multivariable analysis of factors associated with nonadherence to antihypertensive medications among hypertensive patients in a follow-up clinic at Asella Referral and Teaching Hospital, Asella Town, Arsi Zone, Oromia Regional State, Ethiopia, 2020 (*n* = 115).

Variables	Categories	Medications		COR (95% CI)	AOR (95% CI)	*P* value
		Adherence	Nonadherence			
Gender	Male	43 (76.8%)	13 (23.2%)	2.67 (0.94,7.61)	**2.09 (1.93,9.59)**	**0.035**
	Female	53 (89.8%)	6 (10.2%)	1	1	

Age in years	≤45	35 (81.4%)	8 (18.6%)	1.27 (0.47,3.45)	1.55 (0.45,5.29)	0.487
	>45	61 (84.7%)	11 (15.3%)	1	**1**	

Marital status	Unmarried	52 (91.2%)	5 (8.8%)	1	1	
	Married	44 (75.9%)	14 (24.1%)	3.31 (1.11,9.91)	**4.22 (1.29,13.76)**	**0.017**

Educational status	No formal education	30 (83.3%)	6 (16.7%)	1.02 (0.35,2.93)	0.95 (0.27,3.36)	0.936
	Formal education	66 (83.5%)	13 (16.5%)	1	1	

Occupation	Unemployed	44 (80%)	11 (20%)	1.63 (0.60,4.39)	2.03 (0.54,7.66)	0.296
	Employed	52 (86.7%)	8 (13.3%)	1	1	

Average monthly income	≤2500 ETB	59 (80.8%)	14 (19.2%)	1.76 (0.58,5.28)	**1.58 (1.09,7.08)**	**0.041**
	>2500 ETB	37 (88.1%)	5 (11.9%)	1	1	

Duration since diagnosis	≤5 years	63 (84%)	12 (16%)	0.89 (0.32,2.49)	0.84 (0.27,2.66)	0.769
	>5 years	33 (82.5%)	7 (17.5%)	1	1	

Presence of comorbidities	Yes	16 (69.6%)	7 (30.4%)	2.92 (0.99,8.55)	2.76 (0.83,9.15)	0.097
	No	80 (87%)	12 (13%)	1	1	

## Data Availability

The data used to support the findings of this study are available upon request from the corresponding author.
